# School engagement of children in early grades: Psychometric, and gender comparisons

**DOI:** 10.1371/journal.pone.0225542

**Published:** 2019-11-27

**Authors:** Morteza Charkhabi, Evgeny Khalezov, Tatyana Kotova, Julien S Baker, Frédéric Dutheil, Marie Arsalidou

**Affiliations:** 1 National Research University Higher School of Economics, Moscow, Russia; 2 Université Clermont Auvergne, CNRS, LaPSCo, Physiological and Psychosocial Stress, Clermont-Ferrand, France; 3 Russian Academy for National Economy and Public Administration, Moscow, Russia; 4 Centre for Health and Exercise Science Research, Department of Sport, Physical Education and Health, Hong Kong Baptist University, Kowloon Tong, Hong Kong; 5 Université Clermont Auvergne, CNRS, LaPSCo, Physiological and Psychosocial Stress, University Hospital of Clermont-Ferrand, CHU Clermont-Ferrand, Preventive and Occupational Medicine, WittyFit, Clermont-Ferrand, France; 6 York University, Toronto, Ontario, Canada; University of Lleida, SPAIN

## Abstract

School engagement reflects the degree to which students are invested, motivated and willing to participate in learning at their school and this relates to future academic and professional success. Although school engagement is a primary factor predicting educational dropout or successful school completion in Europe and North America, little is known about school engagement factors in non-English speaking countries. We adapted a 15-item school engagement scale and assessed validity and reliability of the Russian translation on a sample of Russian school-aged children (*N* = 537, 6–12 years, 46% females) who attended at public schools in Moscow. Results of the final factorial structure that included emotional, cognitive and behavioral components were selected based on its excellent fit indices and principles of parsimony. Component results show that the emotional component has the highest internal consistency and the behavioral component has the lowest. Although, all components are significantly interrelated, we observed no gender differences and no significant correlation with age. Theoretically, our data agree with the notion that children’s emotional engagement in schools sets the foundation for learning, participating and succeeding in school activities. Practically, the proposed scale in Russian can be used in educational and clinical settings with Russian speaking children.

## Introduction

School engagement is highly related to educational dropout or successful school completion [1, 2]. It is defined as the extent to which students are committed to school and motivated to learn in a school setting [3, 4]. According to 2013 estimates of the UNESCO Institute of Statistics [5] 86.3% of people worldwide aged 15 and older are literate (Russia 99.7%; Europe 99.13%). Literacy rates are related to scholastic success and school engagement that is driven by emotional, cognitive, and behavioral investment students apply for learning [6]. There is general agreement concerning the multidimensional nature of school engagement in North America and Europe as a meta-construct with two to three main dimensions: behavioral, emotional and cognitive [6, 7, 8]. Despite the high literacy rates in Russia, little is known on how children engage in the classrooms. Critically, we are not aware of any scales in Russian speaking countries that assess the degree of school engagement using this tri-factor approach (i.e., behavioral, emotional and cognitive). Our study adapted a 15-item school engagement scale proposed in English by Fredricks, Blumenfeld, Friedel and Paris [9] to Russian and examined linguistic accuracy, validation and reliability in school aged children.

Various researchers have tried to define different dimensions of school engagement. For example, some define behavioral engagement as positive conduct, putting forth effort, and participation in school activities [10, 11]. Emotional or affective engagement is conceptualized as an interest in the school, identification with the school, school belonging, and positive attitude about learning in the school [12, 13]. Although these behavioral and emotional dimensions are preliminarily viewed as the main components of school engagement, recent studies have incorporated a third component, cognitive engagement [6, 8]. Cognitive engagement is defined as self-regulation, learning goals, and investment in learning [6, 8]. Classification of the three dimensions is seated in those theories proposing to provide students with fundamental needs such as autonomy, competence and relatedness leading to personal and academic growth [14, 15].

School engagement is a popular topic in education and it has been extensively studied in Europe and North America. Despite concerns of student disengagement linked to drop out rates, studies show that school engagement is linked with various positive outcomes. Specifically, a school engagement scale developed by Fredericks et al. [9] has been adapted and used in countries of Europe, North America and Asia (Table 1). For example, Dolzan, Sartori, Charkhabi, and De Paola [16] found that higher school engagement is related to higher levels of student self-efficacy and lower levels of health-risk behaviors among Italian high school students. In a similar study, Veiga [17] tested the relation between school engagement and scholastic performance; a high positive association was observed between school engagement and grades on mathematics among Portuguese students.

**Table 1:** A selected review of studies that used or adapted the school engagement scale by Fredricks et al [9].

First Author	Year	Sample	Males	Grade	Age range	Relation with age	Relation with gender	Strongest component	Country
Teuscher [43]	2018	220	115	7^th^ and 9^th^	14.7*	ns	ns	n/r	Switzerland
Sanyal [44]	2017	300	75	8^th^ to 10^th^	13–16	n/r	ns	n/r	India
Mai [21]	2015	460	199	n/r	12–17	n/r	n/r	Emotional	Malaysia
Ramos-Diaz [47]	2016	1543	728	n/r	12–18	sig	sig	Emotional	Spain
Dolzan [16]	2015	250	64	n/r	13–21	n/r	n/r	n/r	Italy
Vazirabadi[45]	2010	362	194	8th	n/r	n/r	ns	n/r	USA
Zahed [48]	2013	360	n/r	6th, 7th, 8th	n/r	n/r	n/r	n/r	Iran
Yusof [49]	2017	1027	596	7th, 8th, 9th	12–19	n/r	n/r	Cognitive	Singapore
Fredricks [42]	2003	991	n/r	3th, 4th, 5th	n/r	sig	sig	Emotional	USA

* Age in years, reported as a mean or range. n/r = not reported, sig = significant, ns = not significant

In the Russian literature, the most thematically relevant research examines children’s motivation to learn. For example, questionnaires exist that evaluate a student’s internal (e.g., self-driven interest in learning) or external (e.g., my parents expect me to learn) motives for learning [18, 19, 20]. Although motivation for learning is an important individual characteristic of children and their incentives for learning, these questionnaires cannot account for the state of the child at the school. The state of the child in the school would include the child’s coping, interest and enjoyment in educational and school activities. We are not aware of any scales in Russian that assess the degree of school engagement distinguishing between behavioral, emotional and cognitive components. With the current study, our main aim is to adapt a questionnaire in Russian that can capture the state of school engagement in children in terms of cognitive, emotional and behavioral factors.

The tri-factor scale presented by Fredericks, Blumenfeld, Friedel, and Paris [9] evaluates behavioral, emotional, and cognitive dimensions of school engagement. It includes questions such as “*I follow the rules at school*”, “*I feel excited by the work in school*” and “*I check my schoolwork for mistakes*”. This scale has been validated and used in other non-English speaking countries. The reason this scale has been widely accepted and used is that it shows good psychometrical properties to indicate school engagement regardless of the cultural and social norms of the host country [e.g., 16, 7, 21]. For this study, we translated and linguistically validated the scale by Fredericks, Blumenfeld, Friedel, and Paris [9] to study its psychometric properties in the Russian context.

## Method

### Participants

This study included school children aged between 6 and 12 years attending publicly funded schools in Moscow. According to the Russian Ministry of Education in 2017 there were a total of 625 publicly funded schools. School education is provided on the basis of a unified system with prescribed course subjects, topics and appointed textbooks in the Russian language. We randomly chose and contacted 25 of those schools, five of which were interested to participate in a larger study that included assessment of school engagement. The five schools that accepted to participate were distributed throughout the official schools ranking in Moscow as posted by the Ministry of Education, thus we are confident in terms of demographic representation of Russian children. Upon approval from the Principal, teachers were introduced to the study by the school psychologist and our researchers. Teachers distributed consent forms to children and only children who returned signed written consent forms with their agreement to participate in the study were invited to participate. Children provided verbal assent. Data collection occurred between September 2017 and May 2018. All Participants were children in grades 1, 2, 3 and 4 whose parents provided signed written consent forms. We received 550 signed written consent forms from parents (participation rate was ~ 36%). Data from 13 students were discarded because the children did not appropriately answer the questions or stopped participating. The final sample (*n* = 537) mean age 9.34±1.03 included 46% girls. According to Kline [22] the minimum sample size required to test the validity of a measurement tool using confirmatory factor analysis (CFA) is 200, or 5 times the number of parameters. In doing so, for testing the first model we had 33 parameters (15 variances, 15 factor loadings, and 3 correlations), which required at least 165 participants (33 × 5 = 165). For testing the second model we had 35 parameters (17 variances, 15 factor loadings, and 3 correlations) and thus we required 175 participants (35 × 5 = 175). For testing the third model, we had 31 parameters (15 variances, 13 factor loadings, and 3 correlations) and thus we required 155 participants (31 × 5 = 155). Overall, our sample satisfied these minimum requirements. The local Research Ethics Board at the National Research University Higher School of Economics of Moscow, Russia approved all methods and procedures including filling and signing writing consent form by parents of students and analyzing the data anonymously.

### Measures

The original school engagement scale in English [9] consists of 15 items divided between three sub-scales: behavioral engagement (4 items), emotional engagement (6 items), and cognitive engagement (5 items; Table 2). Responses are rated using a 5-point Likert scale (1 = never, 2 = on occasion, 3 = some of the time, 4 = most of the time, 5 = all of the time). Total scores range from 15 to 75, and higher scores indicate greater school engagement. Two items of this scale are scored in reverse (items 4 and 6; Table 2). The Russian version of this scale we called the scale of School Engagement in Children—Russian (SEC-RU). SEC-RU was developed based on the translation and cultural adaptation processes recommended by the World Health Organization [23]. More specifically, a bilingual university PhD candidate primarily translated the scale into Russian, and three additional bilingual university professors with a background in developmental psychology checked the back translation of this scale as well as the face and content validity of the translated version of the original scale [9]. According to Lynn [24], the appropriate number of judges for checking the content validity is estimated between three and ten. All items and their translation are on Table 2. Examples include: “*I follow the rules at school*” for behavioral engagement, “*I feel excited by the work in school*” for emotional engagement and “*I try to watch TV shows about things we are doing at school*” for cognitive engagement.

**Table 2:** Item factor loadings for all three models and descriptive statistics for all items (*n* = 537).

		Factor loadings			Descriptive Statistics			
	Scale Items	Initial model	Revised model	Final model	M	SD	Min	Max
Behavioral								
	1. I pay attention in class. Я внимателен в классе.	.56	.56	.55	3.76	.93	1	5
	2. When I am in class I just act as if I am working. На уроках я делаю задания.	.44	.44	.44	4.56	.83	1	5
	3. I follow the rules at school. Я соблюдаю школьные правила.	.51	.51	.48	4.12	.97	1	5
	4. I get in trouble in school.* Я могу получить замечание в школе из-за поведения.	.36	.36	-	3.76	1.06	1	5
**Emotional**								
	5. I feel happy in school. В школе мне хорошо.	.67	.70	.70	4.18	1.04	1	5
	6. I feel bored in school.* В школе мне скучно.	.77	.76	.76	4.12	1.08	1	5
	7. I feel excited by the work in school. Мне нравится учеба в школе.	.53	.53	.52	3.73	1.22	1	5
	8. I like being in school. Мне нравится быть в школе.	.82	.80	.81	3.99	1.17	1	5
	9. I am interested in the work at school. Мне интересно выполнять задания в классе.	.68	.71	.70	3.95	1.11	1	5
	10. My classroom is a fun place to be. Мне весело в классе.	.53	.53	.53	4.02	1.18	1	5
**Cognitive**								
	11. I study at home even when I don’t have a test.Я занимаюсь дома, даже когда знаю, что никто не будет проверять меня.	.50	.50	.51	3.61	1.40	1	5
	12. I try to watch TV shows about things we are doing at school. Я смотрю передачи, связанные с учебой.	.58	.51	.51	2.49	1.26	1	5
	13. I check my schoolwork for mistakes. После того, как я сделал мое домашнее задание, я проверяю нет ли ошибок.	.64	.66	.64	3.81	1.34	1	5
	14. I read extra books to learn more about things we do in school. Мне нравится читать книги про то, что мы проходим в школе.	.68	.63	.62	3.21	1.31	1	5
	15. If I don’t know what a word means when I am reading, I do something to figure it out, like look it up in the dictionary or ask someone. Когда я читаю, я спрашиваю у взрослых, что означают незнакомые слова.	.35	.36	-	3.98	1.20	1	5

Note: Behavioral: items 1–4, Emotional: items 5–10, Cognitive: items 11–15. *Reversed items

### Procedure

Students were asked to individually complete the questionnaire in a quiet room in their school. They had enough time to read the questions and in case they could not understand a question they were encouraged to raise their hand and ask the experimenter. On a 5-point Likert scale they marked a box that corresponded to their response. At the end of the test, each student received a small gift (e.g., pencil, eraser). Each questionnaire was coded and tabulated for data analysis.

### Data analyses

We used IBM SPSS-22 and IBM AMOS-22 to analyze the data. IBM SPSS-22 was used to calculate descriptive statistics (e.g., means and standard deviations), internal reliability of the scale as a whole and each component separately. IBM AMOS-22 was used to assess the factorial validity of the scale using confirmatory factor analysis (CFA). Construct validity was determined based on the factorial validity of the sub-scales, and a model-fit test of the scale structure using CFA.

## Results

### Descriptive statistics

Table 3 shows descriptive statistics of each component and overall scores as a function of age group.

**Table 3:** Descriptive statistics for each component and an overall score of school engagement (*n* = 537).

	Behavioral component		Emotional component		Cognitive component		Overall score	
	Mean	SD	Mean	SD	Mean	SD	Mean	SD
Grade 1 (n = 48)	4.01	0.82	4.18	0.70	3.57	0.81	3.89	0.65
Grade 2 (n = 173)	4.01	0.63	4.05	0.84	3.58	0.87	3.83	0.69
Grade 3 (n = 161)	4.11	0.60	4.02	0.86	3.51	0.81	3.83	0.65
Grade 4 (n = 155)	4.06	0.55	3.92	0.76	3.49	0.70	3.77	0.58
Total sample (n = 537)	4.05	0.62	4.01	0.81	3.53	0.80	3.82	0.64

Correlational methods and analyses of variance.

Table 4 shows correlations among components of school engagement and age. Although there is a reverse relation among the components of school engagement and age, these relations were not statistically significant, thus controlling for age minimally affected the correlations among school engagement components. Analyses of variance were used to examine differences in performance between boys and girls per age group; no statistically significant differences were observed.

**Table 4:** Correlations among components and age (n = 537).

	Age	Behavioral engagement	Emotional engagement	Cognitive engagement	Overall school engagement
Age	-	- .019	-.046	-.033	-.044
Behavioral engagement		-	.448**	.451**	-
Emotional engagement	-	.447**	-	.599**	-
Cognitive engagement	-	.451**	.598 **	-	-
Overall school engagement	-	-	-	-	-

Note: Bivariate Pearson’s r correlations are above the diagonal; Partial Pearson’s r correlations controlling for age are below the diagonal. **p<0.01 (2-tailed)

### Psychometric characteristics of the SEC-RU scale

We used *confirmatory factor analysis* (CFA) to find the best factorial structure of the school engagement scale using AMOS-22 [25]. CFA was run using the maximum-likelihood method. Because a fit index reflects only a specific aspect of model fit, a single good value cannot provide enough evidence for a good fit [26, 27, 28]. Thus, the goodness-of-fit of the models was estimated by means of several indices that were interpreted relatively to each other [29]: Chi-square statistic (χ2); Comparative fit index (CFI); Tucker-Lewis Index (TLI); Root mean square error of approximation (RMSEA); Standardized Root Mean Square Residual (SRMR); Normed Fit Index (NFI). For the RMSEA and SRMR, values smaller than 0.08 indicate good fit [30, 29, 27].Values greater or equal to 0.90 on the CFI, the TLI and the NFI indicate good fit [31]. Weston and Gore [32] explained that the Chi-squared statistic is sensitive to sample size and tests whether the model shows an exact fit to the data, a finding that is rare. The Chi-squared statistic should not be used as a direct indication for the goodness-of-fit of a model. Hence, we used the Chi-squared statistic to compare competing models [32].

### Validity of the SEC-RU scale

**Fig 1:**
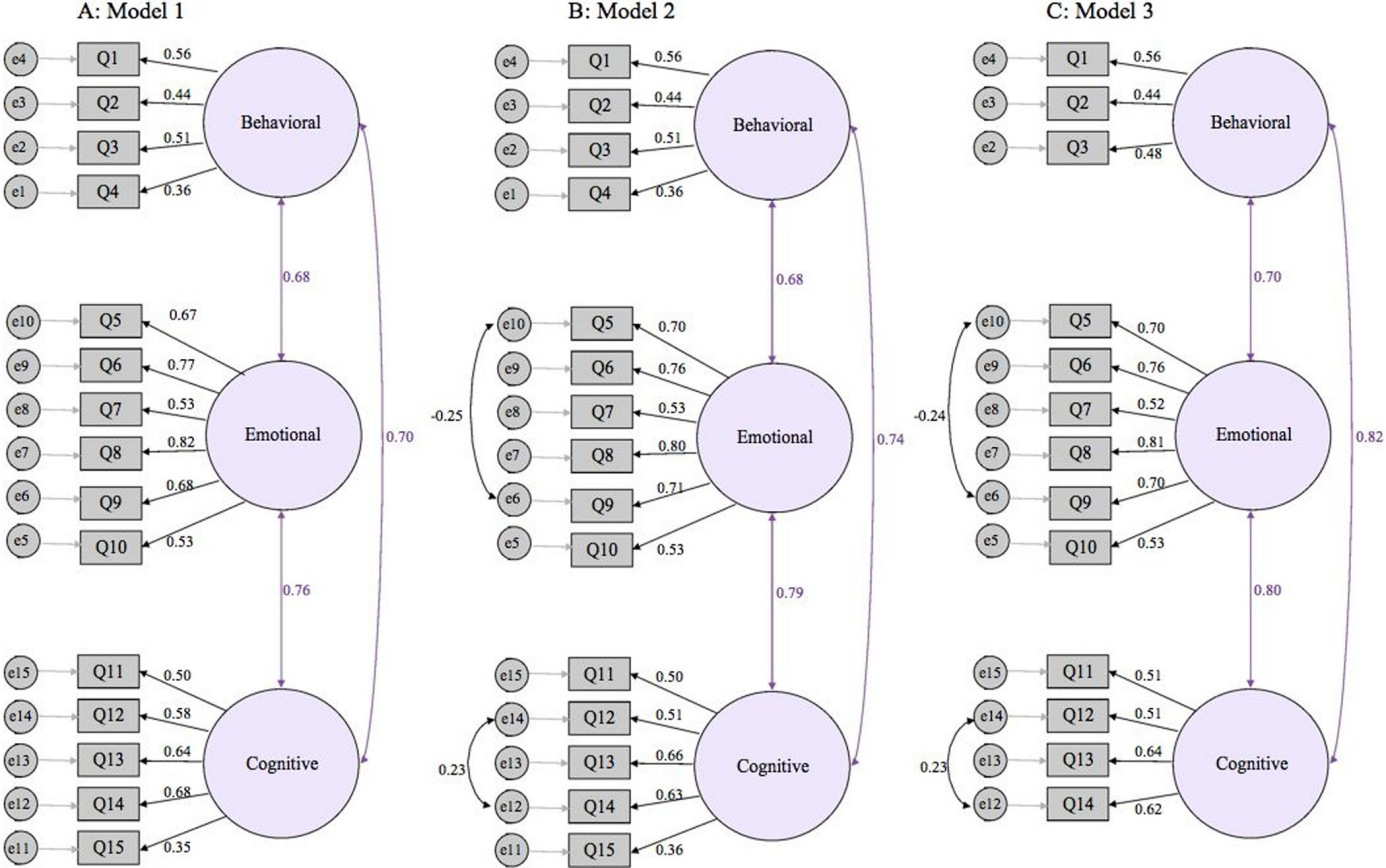
Standardized regression weights in (A) Model 1: an initial confirmatory factor analysis model of the 15-item SEC-RU scale, (B) Model 2: a revised confirmatory factor analysis model of the 15-item SEC-RU scale, and (C) Model 3: a final confirmatory factor analysis model of a 13-item SEC-RU scale.

Three models were tested and compared using CFA on the total sample size (*N* = 537; Table 5). First, an initial model with 15 items loading on three factors (behavioral, emotional and cognitive engagement) was estimated (Fig 1). In this Model, the first factor (behavioral engagement) contained four observed variables, the second factor (emotional engagement) contained six observed variables, and the third factor (cognitive engagement) contained five observed variables. This model showed moderate fit indices (χ2/df = 2.77, SRMR = .05, CFI = .93, TLI = .91, NFI = .89, and RMSEA = .06) and two low factor loadings (i.e., ≤.40).

**Table 5:** Goodness-of-fit indicators of SEC-RU (*n* = 537).

Models	χ2/df	CFI	TLI	NFI	RMSEA*	SRMR	ΔCFI	ΔRMSEA
1. Three factors without covariance, 15 items	2.77	.93	.91	.89	.06	.05	-	-
2. Three factors with covariance, 15 items	2.36	.94	.93	.91	.05	.04	-.01	.01
3. Three factors with covariance, 13 items	2.16	.96	.95	.94	.04	.03	-.02	.01

* CI = 95% confidence interval, *Note*: χ^2^ = chi-square goodness of fit statistic; *df* = degrees of freedom; CFI = Comparative Fit Index; TLI = Tucker Lewis Index; RMSEA = Root-Mean-Square Error of Approximation; SRMR = standardized RMR, root mean square residual; NFI = normed fit index.

To enhance the model fit indices, we revised Model 1 and substituted it with a three-dimensional model that included errors covariates for (a) items 5 and 9 in the emotional component and (b) items 12 and 14 in the cognitive component (Fig 1, Model 2). According to modification indices in the CFA outcome, items 5 and 9, and items 12 and 14 showed the highest common variance, thus error variance for these two pairs were correlated in Model 2.The decision was taken to account for shared variance associated with errors terms of these components. Typically, decisions to correlate error variance rely on high common variance and whether their association significantly enhances the model fit indexes. Specifically, correlating error terms with high covariance can improve the reliability of a latent construct, as measured via goodness of fit indices [33]. We should mention that correlating error variances should be done skeptically, with the consideration that correlating error variances does not hurt the parsimonious aspect of the model in terms of fit indices [33], as is the case with our parsimonious model. Model 2 indices showed noticeable improvements (χ2/df = 2.36, SRMR = .04, CFI = .94, TLI = .93, NFI = .91, and RMSEA = .05), with the exception of two items (4 and 15) with low factor loadings (i.e., ≤ .40).

A third model (Fig 1, Model 3) implemented further model improvements by discarding those items that showed low factor loadings (items 4 and 15). Model 3 thus included three observed variables for the first factor (behavioral engagement), six observed variables for the second factor (emotional engagement), and four observed variables for the third factor (cognitive engagement). We found noticeable improvement in all the fit indices for Model 3 (χ2/df = 2.16, SRMR = .03, CFI = .96, TLI = .95, NFI = .94, and RMSEA = .04). The total number of items in the final model was 13. The standard factor loading of all items ranged from .44 to .81 and they were all significantly different from zero. Fit indices for all three nested models are tabulated in Table 5 and are found to have excellent fit. On the basis of the parsimony principle, we decided to choose Model 3 as the final model and to use it for further analyses.

### Reliability of the SEC-RU scale

Reliability is defined as the degree to which the measurement tool produces stable and consistent results [34]. We applied Cronbach’s alpha to assess the internal reliability of the scale. According to Hinton, Brownlow, McMurray, and Cozens [35] Cronbach’s Alpha values of >0.90, 0.70–0.90, 0.50–0.70, <0.50, correspond to ratings of excellent, high, moderate and low internal reliability, respectively. Following these guidelines, we find high internal reliability for the emotional engagement component (α = .82), a moderate internal reliability for the cognitive engagement component (α = .69; more than .70 is considered high internal reliability) and a low internal reliability for the behavioral engagement component (α = .49; more than .50 is considered moderate internal reliability). In addition, we find high internal reliability for the whole scale (α = .85). Table 6 provides a synopsis the internal reliability of the scale and its components separately. The emotional component shows the highest internal reliability, whereas behavioral engagement shows the lowest reliability.

**Table 6:** Reliability statistics of the finalized version of SEC-RU (*n* = 537).

Components	Cronbach's Alpha	Number of items	Outcome
Behavioral engagement	.491	3	Low internal reliability
Emotional engagement	.822	6	High internal reliability
Cognitive engagement	.690	4	Moderate internal reliability
Overall	.850	13	High internal reliability

## Discussion

According to the world economic forum an estimated 65% of children who are now entering elementary school will graduate to work on completely new occupations that do not exist today [36]. A child’s future professional success is related to the degree of school engagement. Although school engagement trends in English speaking countries show a school engagement rates decreasing with age; school engagement in Russian schools remain unexplored. We adapted a school engagement scale from English [9] and translated items with linguistic and cultural considerations to Russian. This is the first study that examined a tri-factor school engagement scale in elementary school children from public schools in Moscow. We highlight three main findings: (a) the translated scale, SEC-RU has high psychometric characteristics for measuring school engagement in children who speak Russian, (b) the strongest component of the school engagement scale is the emotional, consistent with past reports and (c) school engagement does not show significant decreases as a function of age, in contrast with past reports.

A main purpose of our study is to validate psychometric properties of a school engagement scale in Russian. We examine three models. The first two models use all 15 items and show very good model-fit indices and good factor loadings on 13 out of 15 items. The two items that have sub-threshold loadings are “I get in trouble in school” in the behavioral component and “If I do not know what a word means when I am reading, I do something to figure it out, like look it up in the dictionary or ask someone”. The final model includes 13 items with the highest factor loadings and shows good to high model-fit on all indices of the three components of school engagement. The highest factor loading was observed to the emotional component whereas, the lowest factor loading was found in the behavioral component. The behavioral component evaluates the child’s conduct at the school, such as adhering to classroom norms, which may be less variable in the younger children we have tested. Emotional engagement, however, addresses a child’s affective reactions to the school and to the teacher, which shows the strongest factor loading. This finding is consistent with results we obtain from reliability statistics. Past studies also showed the emotional component having the highest factor loading [9, 37], suggesting that emotional factors are the strongest predictors of school engagement.

As the findings reveal, item reductions in the behavioral and cognitive components increase the fit indices, however, it also decrease the overall internal reliability of the scale as well as the internal reliability of behavioral component (from .53 to .49), which is not a favored outcome. As such, we opted to discard the two items with low factor loadings (< .40) from both components to improve the fit indices. After these items are constrained, the model indices gain significant improvements. Furthermore, in Model 3 all internal reliabilities for the behavioral, emotional and cognitive engagement (range: 0.49 to 0.82) are adequate to conclude scale reliability. Notably, reliability of the emotional component is strongest, followed by the cognitive component and behavioral component, in that order. This is similar to the findings of Fredricks et al [9] who found high reliability for emotional engagement and low reliability for behavioral engagement. Lower reliability for the behavioral and cognitive engagement could be related to fewer items after dropping two items from these components. Therefore, we conclude that the final version of this scale containing three components, 13 items and two constrains has the best psychometrical characteristics to be used with Russian speaking children. This finding needs to be replicated by future studies.

Our study is also one of the first to examine children’s tri-factor school engagement in primary school grades in Russia and worldwide (Table 1). We examine early school grades, starting with children aged 6 years. Although all factors are highly inter-correlated, no statistically significant correlation was observed between school engagement and age. Previous studies found that school engagement decreases as a function of school grade [38, 39]. Being less engaged with the school in older children might be related to transition from childhood to adolescence. This transition is aligned with psychological changes such as the need to receive more freedom and less control [40], thus report less school engagement to protect their emerging identity as adolescents. This lack of statistically significant correlation with age in our data may be understood in three ways. First, no age cohort effects were observed in this study and future longitudinal studies may consider it. A second alternative is related to the sample range. Although our study used four age groups to measure this construct, our sample focused on primary school grades which included children that were younger than previous studies [4, 16, 41]. A third alternative explanation may not be related to students but to the culture of the schools environments. If schools in Russia provide a uniform learning environment for students, they might be uniformly motivated to get engaged in school activities and show their interest to school. Such socio-cultural differences in education systems may form targets for future research to better understand the relation of age and school engagement among cultures. No significant gender differences were observed in our sample. Although some previous studies reported gender effects in school engagement [38, 42], other studies do not [43, 44, 45], yet most studies do not report statistics on gender differences (Table 1).

### Theoretical implications

School engagement is a well-established construct that relies on various components that condense to cognitive, emotional and behavioral factors. Theoretically, school engagement has a bidirectional relations with many factors associated with social context, classroom quality, school environment, academic success and well-being [46]. Indeed empirical findings show a strong relation with academic success and well-being. Critically, the model by Upadyaya and Salmela-Aro [46], presents a component that incorporates developmental stages that is less well understood. Developmental stages related with school engagement are less well understood because most studies focus on school engagement in adolescence (see Table 1). Our data suggest that a tri-factor model also defines school engagement in younger school-age children, and that the emotional component remains the strongest, same as in older children (Table 1). In this respect our study replicates and extents previous findings in a culturally different setting. In contrast with adolescents, younger children show consistent school engagement without a negative relation between school engagement and age on any of the components. The exact time when these relations materialize remains to be investigated and warrants further research with larges age ranges.

### Practical implications

School engagement is a multifaceted construct that has implications to both research and practice [8]. Engaged children become professionally successful and well-adjusted adults [46]. Professionally successful adults become the cognitive capital of a nation. Thus, it is in the interest of advance nations to better understand school engagement. Practically, we provide the first indices on school engagement in children as young as six years, which is important for future studies in establishing the trajectory of school engagement across development. Further, we provide a psychometrically validated scale in Russian, which is derived from an established scale already used by various other non-English speaking countries. This contribution is fundamental for studies across different language backgrounds and cultures; as well as among the 15 counties in the world who have Russian as a main language. Furthermore, clinical or educational psychologists can use this scale as a tool for detecting underlying factors associated with difficulties in schools and drop-out tendencies.

## Conclusion

We tested the psychometric properties of a tri-factor scale examining school engagement in elementary school children. The final 13-item version of the SEC-RU scale showed adequate validity and reliability indices in the Russian sample. The three outstanding advantages of the SEC-RU are (a) it is short and parsimonious for measuring the three generally accepted dimensions of the student engagement in school, (b) it is self-administrated to children as young as six years and (c) it is useful for group testing in educational and school settings. Overall, this short scale fulfills psychometric characteristics for school engagement measurements in Russian and provides a fast, easy, and useful instrument for clinical practitioners and educational settings.

## Supporting information

S1 Dataset (XLSX)
