# Correction: School engagement of children in early grades: Psychometric, and gender comparisons

**DOI:** 10.1371/journal.pone.0228605

**Published:** 2020-01-28

**Authors:** Morteza Charkhabi, Evgeny Khalezov, Tatyana Kotova, Julien S Baker, Frédéric Dutheil, Marie Arsalidou

The fourth author’s initials appear incorrectly in the citation. The correct citation is: Charkhabi M, Khalezov E, Kotova T, Baker JS, Dutheil F, Arsalidou M (2019) School engagement of children in early grades: Psychometric, and gender comparisons. PLoS ONE 14(11): e0225542. https://doi.org/10.1371/journal.pone.0225542.
